# High prevalence of meniscal ramp lesions in anterior cruciate ligament injuries

**DOI:** 10.1007/s00167-022-07135-8

**Published:** 2022-08-31

**Authors:** Riccardo Cristiani, Fabian van de Bunt, Joanna Kvist, Anders Stålman

**Affiliations:** 1 Department of Molecular Medicine and Surgery, Stockholm Sports Trauma Research Center, Karolinska Institutet, Stockholm, Sweden; 2 Capio Artro Clinic, FIFA Medical Centre of Excellence, Sophiahemmet Hospital, Valhallavägen 91, 11486 Stockholm, Sweden; 3 Division of Radiology, Department of Clinical Science, Karolinska University Hospital, Stockholm, Sweden; 4 Department of Health, Medicine and Caring Sciences, Division of Prevention, Rehabilitation and Community Medicine, Unit of Physiotherapy, Linköping University, Linköping, Sweden

**Keywords:** Ramp lesion, Meniscus, Anterior cruciate ligament, ACL, Magnetic resonance imaging, MRI, Bone bruise, Knee, Association

## Abstract

**Purpose:**

To evaluate the prevalence of and factors associated with meniscal ramp lesions on magnetic resonance imaging (MRI) in patients with anterior cruciate ligament (ACL) injuries.

**Methods:**

Data from the Natural Corollaries and Recovery after ACL injury multicentre longitudinal cohort study (NACOX) were analysed. Only patients who underwent MRI were included in this study. All MRI scans were reviewed by an orthopaedic knee surgeon and a musculoskeletal radiologist. The patients were divided into two groups, those with and without ramp lesions according to MRI findings. Univariable and stepwise forward multiple logistic regression analyses were used to evaluate patient characteristics (age, gender, body mass index, pre-injury Tegner activity level, activity at injury) and concomitant injuries on MRI (lateral meniscus, medial collateral ligament [MCL], isolated deep MCL, lateral collateral ligament, pivot-shift-type bone bruising, posteromedial tibial [PMT] bone bruising, medial femoral condyle bone bruising, lateral femoral condyle [LFC] impaction and a Segond fracture) associated with the presence of meniscal ramp lesions.

**Results:**

A total of 253 patients (52.2% males) with a mean age of 25.4 ± 7.1 years were included. The overall prevalence of meniscal ramp lesions was 39.5% (100/253). Univariate analyses showed that contact sports at ACL injury, pivot-shift-type bone bruising, PMT bone bruising, LFC impaction and the presence of a Segond fracture increased the odds of having a meniscal ramp lesion. Stepwise forward multiple logistic regression analysis revealed that the presence of a meniscal ramp lesion was associated with contact sports at ACL injury [odds ratio (OR) 2.50; 95% confidence intervals (CI) 1.32–4.72; *P* = 0.005], pivot-shift-type bone bruising (OR 1.29; 95% CI 1.01–1.67; *P* = 0.04), PMT bone bruising (OR 4.62; 95% CI 2.61–8.19; *P* < 0.001) and the presence of a Segond fracture (OR 4.38; 95% CI 1.40–13.68; *P* = 0.001).

**Conclusion:**

The overall prevalence of meniscal ramp lesions in patients with ACL injuries was high (39.5%). Contact sports at ACL injury, pivot-shift-type bone bruising, PMT bone bruising and the presence of a Segond fracture on MRI were associated with meniscal ramp lesions. Given their high prevalence, meniscal ramp lesions should be systematically searched for on MRI in patients with ACL injuries. Knowledge of the factors associated with meniscal ramp lesions may facilitate their diagnosis, raising surgeons’ and radiologists’ suspicion of these tears.

**Level of evidence:**

III.

## Introduction

Meniscal ramp lesions are peripheral tears of the medial meniscus (MM) involving the meniscocapsular ligament, meniscotibial ligament and/or the red-red zone of the posterior horn, in the setting of an anterior cruciate ligament (ACL) tear [13, 14, 40]. Previous studies have shown that meniscal ramp lesions are associated with increased anterior and rotational knee laxity in the ACL-deficient knee [1, 12, 29, 30, 37] and that only their repair restores knee biomechanics [1, 12, 37]. Moreover, patients with meniscal ramp lesions exhibit accelerated cartilage degeneration in the medial compartment in comparison with controls [15]. It is therefore essential to recognise these injuries.

The literature is inconsistent regarding the prevalence of meniscal ramp lesions. Previous studies have reported a wide variation in the prevalence of meniscal ramp lesions diagnosed at the time of ACL reconstruction (ACLR) [6, 11, 26, 36].

Even though direct arthroscopic visualisation is generally regarded as the gold standard for diagnosing ramp lesions [6, 26, 36], several ramp lesions might be missed intraoperatively due to their difficult visualisation and probing through the anterolateral and anteromedial portals [36].

Magnetic resonance imaging (MRI) is regarded as the best imaging modality to diagnose meniscal ramp lesions [10, 14, 43, 44], even though its accuracy has been questioned due to the varying sensitivity reported in previous studies [2, 11, 19, 41]. In their recent systematic review and meta-analysis, Koo et al. [21] reported that MRI has high specificity (94%) but moderate sensitivity (71%) for diagnosing ramp lesions. It should, however, be noted that studies evaluating MRI accuracy have used different and limited pathological signs to define ramp lesions [2, 11, 20, 25, 41, 43]. In some studies [6, 28, 40] MRI criteria were not even reported. In a recent study, using an extension of Thaunat’s classification [39], Greif et al. [14] described seven different types of meniscal ramp lesion together with their MRI appearance. Failure to consider the MRI appearance of the different types of meniscal ramp lesion may have been responsible for an underestimation of the true prevalence of these injuries and the reported reduced sensitivity of this imaging modality in the literature.

An awareness of the prevalence and appearance of the different types of meniscal ramp lesion on MRI may improve the diagnosis of these injuries and would alert the orthopaedic surgeon to focus particularly on the posteromedial ramp area during ACLR. In addition, detailed knowledge of potential epidemiological factors and injuries on MRI associated with the presence of meniscal ramp lesions may further facilitate the diagnosis of these injuries by raising surgeons’ and radiologists’ suspicion of these important tears.

The primary purpose of this study was to evaluate the prevalence of meniscal ramp lesions in patients with ACL injuries, using well-defined MRI pathological signs [14]. Another purpose was to investigate epidemiological factors and injuries on MRI associated with the presence of meniscal ramp lesions. It was hypothesised that the prevalence of meniscal ramp lesions was high and that younger age, contact sports at ACL injury and the presence of posteromedial tibial (PMT) bone bruising or a Segond fracture would be associated with the presence of meniscal ramp lesions.

## Materials and methods

Data were extracted from a prospectively collected patient database. This study is part of the Natural Corollaries and Recovery after ACL injury study [NACOX] [24]. Ethical approval was obtained from the regional ethics committee in Linköping, Sweden (Dnr 2016/44-31 and 2017/221-32). All the patients signed informed consent to participate. Patients were recruited between 2016 and 2018, from six orthopaedic clinics in Sweden. The inclusion criteria were ACL injury sustained no more than six weeks prior to presentation and age between 15 and 40 years at the time of injury. Patients were excluded if they had had previous ACL injury/surgery on the same knee, fractures that required separate treatment, an inability to understand the written or spoken Swedish language, cognitive impairments, other illnesses or injuries that impaired function (e.g. fibromyalgia, rheumatic diseases and other diagnoses associated with chronic pain) [24]. ACL ruptures were diagnosed by an orthopaedic surgeon and were, if needed, verified by MRI. For the purposes of this study, only patients who underwent MRI scans were included.

### Data collection

The assessed patient characteristics were age at injury, gender, body mass index (BMI), pre-injury Tegner activity level [38] and activity at ACL injury. Age at injury was dichotomised into unbiased classes close to the median (< 25 years or ≥ 25 years). The BMI was dichotomised at 25, as a BMI of ≥ 25 is regarded as overweight [42]. The pre-injury Tegner activity level was dichotomised as high (≥ 6) or low (< 6). Finally, the activity at ACL injury was dichotomised as contact sports or non-contact sports/other.

### Radiological assessment

The majority (*n* = 209) of the patients underwent MRI scans at two institutions (Capio Artro Clinic, Stockholm, Sweden, and Linköping University Hospital, Linköping, Sweden). The remaining patients underwent MRI scans at the other institutions involved in the NACOX study [24]. The mean time from ACL injury to MRI was 19.6 ± 15.2 days. MRI examinations were performed using a 1.5 (*n* = 115) or 3.0 (*n* = 138) Tesla scanner. The images were acquired in three planes (sagittal, axial and coronal) using T1-weighted, T2-weighted and proton-density (PD) fat saturation sequences. The slice thickness was 3 mm with a 0.3 mm gap. All the MRI scans were independently analysed by an orthopaedic knee surgeon (RC) and a musculoskeletal radiologist (FvdB). In the event of inconsistencies, the examiners assessed the MRI scans together and reached a consensus in a second phase.

The presence of meniscal ramp lesions was best assessed on sagittal images on PD fat saturation or T2-weighted sequences and was defined according to the MRI appearance and classification described by Greif et al. [14]. Seven different subtypes of meniscal ramp lesion were evaluated: type 1, meniscocapsular ligament tear; type 2, partial superior peripheral meniscal horn tear; type 3A, partial inferior peripheral posterior horn meniscal tear; type 3B, meniscotibial ligament tear; type 4A, complete peripheral posterior horn meniscal tear; type 4B, complete meniscocapsular junction tear; type 5, peripheral posterior horn meniscal double tear.

Injuries to the lateral meniscus (LM), medial collateral ligament (MCL), lateral collateral ligament (LCL) and the presence of a Segond fracture were recorded. Injuries to the MCL or LCL were defined as partial rupture/discontinuity with some preserved fibres or complete disruption [31]. Isolated deep MCL injuries were defined as tears of the meniscofemoral and/or meniscotibial ligament with intact superficial MCL on axial images. The presence and location of bone bruising were also evaluated. Bone bruising in the posteromedial tibial (PMT) plateau and medial femoral condyle (MFC) was recorded. Pivot-shift-type bone bruising was defined as the presence of bone marrow oedema in the posterior aspect of the lateral tibial plateau and the midportion of the lateral femoral condyle (LFC) [32]. Finally, the presence of an LFC impaction was defined as an osteochondral depression with an intact or disrupted articular surface [31].

### Statistical analysis

Statistical analysis was performed using the Statistical Package for Social Sciences, SPSS (version 25.0; IBM Corp., Armonk, New York, USA). All the variables were summarised with standard descriptive statistics such as the mean, standard deviations or frequency. Univariable analyses were performed with age (< 25 years vs. ≥ 25 years), gender, BMI (< 25 vs. ≥ 25), pre-injury Tegner activity level (high ≥ 6 vs. low < 6), activity at injury (contact sports vs. non-contact sports/other), LM injury, MCL injury, isolated deep MCL injury, LCL injury, pivot-shift-type bone bruising, PMT bone bruising, MFC bone bruising, LFC impaction and a Segond fracture as independent variables and the presence of meniscal ramp lesions as a dependent variable. A stepwise forward multiple logistic regression analysis was used to identify variables independently associated with meniscal ramp lesions. The independent variables included in the analyses were chosen based on background knowledge and because they were regarded as being clinically relevant to the purpose of the study. The results were reported as odds ratios (OR) with 95% confidence intervals (CI). The level of significance in all analyses was 5% (two tailed).

## Results

### Prevalence of meniscal ramp lesions

**Fig. 1:**
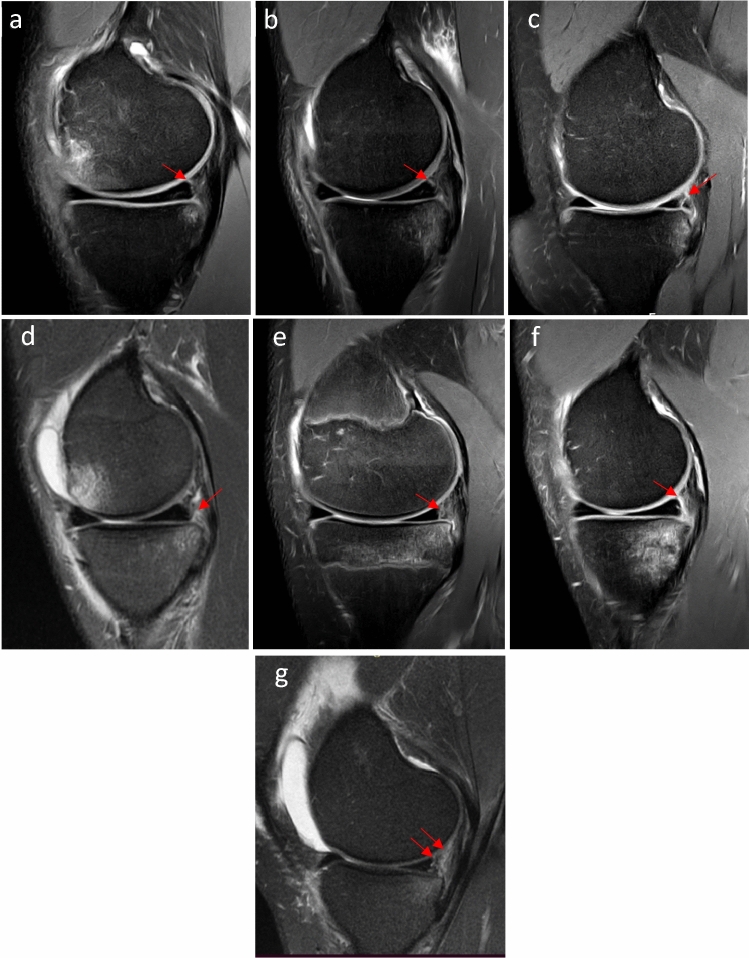
Meniscal ramp lesion subtypes (red arrow) on sagittal proton density fat saturation MRI sequences. **a** Type 1; meniscocapsular ligament tear, as shown by the linear vertical fluid signal reaching the superior articular surface. **b** Type 2; partial superior peripheral meniscal horn tear, as shown by the linear vertical fluid signal reaching the superior articular surface. **c** Type 3A; partial inferior peripheral posterior horn meniscal tear, as shown by the linear oblique fluid signal reaching the inferior articular surface. **d** Type 3B; meniscotibial ligament tear, as shown by the disruption of the ligament with a fluid signal. **e** Type 4A; complete peripheral posterior horn meniscal tear, as shown by the fluid signal extending from the superior to the inferior articular surface. **f** Type 4B; complete meniscocapsular junction tear, as shown by the fluid intensity signal extending from the superior to the inferior articular surface. **g** Type 5; peripheral posterior horn meniscal double tear, as shown by two parallel linear fluid signals extending from the superior to the inferior articular surface. Note that PMT bone bruising is present in all images. MFC bone brusining is present in **a** and **d**. *MFC* medial femoral condyle, *MRI* magnetic resonance imaging, *PMT* posteromedial tibial

A total of 275 patients are included in the NACOX study. Eight patients had a clinical diagnosis of ACL injury and 14 MRIs were not available for the second analysis. Finally, MRIs from 253 patients were analysed in the present study. Overall, meniscal ramp lesions were identified in 100 (39.5%) patients. The subtype distribution was as follows: 13 (13%), type 1 (meniscocapsular ligament tear); 4 (4%), type 2 (partial superior peripheral meniscal horn tear); 7 (7%), type 3A (partial inferior peripheral posterior horn meniscal tear); 7 (7%), type 3B (meniscotibial ligament tear); 20 (20%), type 4A (complete peripheral posterior horn meniscal tear); 43 (43%), type 4B (complete meniscocapsular junction tear); 6 (6%), type 5 (peripheral- posterior horn meniscal double tear) (Fig. 1a–g).

Patient characteristics for both the ramp (*n* = 100) and no-ramp (*n* = 153) groups are summarised in Table 1.

**Table 1:** Patient characteristics and factors associated with a meniscal ramp lesion in univariable logistic regression analysis

	Ramp (*n* = 100)	No-ramp (*n* = 153)	OR (95% CI)	*P* value
Age at injury, years ± SD	25.2 ± 6.8	25.6 ± 7.3		
Age < 25 years	57 (57.0)	75 (49.0)	1.37 (0.83–2.28)	n.s
Age ≥ 25 years	43 (43.0)	78 (51.0)		
Gender				
Male	52 (52.0)	70 (45.8)	1.28 (0.77–2.12)	n.s
Female	48 (48.0)	83 (54.2)		
BMI, mean ± SD	23.4 ± 2.8	24.2 ± 3.5		
< 25	76 (76)	103 (67.8)	1.50 (0.85–2.66)	n.s
≥ 25	24 (24)	50 (32.2)		
Pre-injury Tegner, median (range)	8 (2–9)	7 (2–9)		
High, ≥ 6	69 (69.0)	96 (62.7)	1.32 (0.77–2.25)	n.s
Low, < 6	31 (31.0)	57 (37.3)		
Activity at injury				
Contact sports	76 (76.0)	88 (57.5)	2.33 (1.33–4.09)	0.003
Non-contact sports/other	24 (24.0)	65 (42.5)		
LM injury	30 (30.0)	39 (25.5)	1.25 (0.71–2.19)	n.s
MCL injury	11 (11.0)	31 (20.3)	0.48 (0.23–1.01)	n.s
Isolated deep MCL injury	25 (25.0)	40 (26.1)	0.94 (0.52–1.68)	n.s
LCL injury	3 (3.0)	3 (2.0)	1.54 (0.30–7.81)	n.s
Pivot-shift-type bone bruising	85 (85.0)	98 (64.1)	3.64 (1.72–7.69)	0.0007
PMT bone bruising	61 (61.0)	39 (25.5)	4.57 (2.65–7.86)	< 0.001
MFC bone bruising	16 (16.0)	32 (20.9)	0.72 (0.37–1.40)	n.s
LFC impaction	54 (54.0)	61 (39.9)	1.77 (1.06––2.94)	0.02
Segond fracture	13 (13.0)	6 (3.9)	3.66 (1.34–9.98)	0.01

Data are reported as *n* (%) unless otherwise indicated

*BMI* body mass index, *CI* confidence intervals, *LCL* lateral collateral ligament, *LFC* lateral femoral condyle, *LM* lateral meniscus, *MCL* medial collateral ligament, *MFC* medial femoral condyle, *OR* odds ratio, *PMT* posteromedial tibial

### Univariable analyses

Univariable logistic regression analyses revealed that contact sports at ACL injury (OR 2.33; 95% CI 1.33–4.09; *P* = 0.003), pivot-shift-type bone bruising (OR 3.64; 95% CI 1.72–7.69; *P* = 0.0007), PMT bone bruising (OR 4.57; 95% CI 2.65–7.86; *P* < 0.001), LFC impaction (OR 1.77; 95% CI 1.06–2.94; *P* = 0.02) or a Segond fracture (OR 3.66; 95% CI 1.34–9.98; *P* = 0.01) were associated with the presence of meniscal ramp lesions. Age at an injury, gender, BMI, pre-injury Tegner activity level, LM injury, MCL injury, isolated deep MCL injury, LCL injury and MFC bone bruising were not associated with the presence of meniscal ramp lesions (Table 1).

### Multivariable analysis

Stepwise forward multiple logistic regression analysis revealed that contact sports at ACL injury (OR 2.50; 95% CI 1.32–4.72; *P* = 0.005), pivot-shift-type bone bruising (OR 1.29; 95% CI 1.01–1.67; *P* = 0.04), PMT bone bruising (OR 4.62; 95% CI 2.61–8.19; *P* < 0.001) or a Segond fracture (OR 4.38; 95% CI 1.40–13.68; *P* = 0.001) were significantly associated with the presence of meniscal ramp lesions (Table 2).

**Table 2:** Factors associated with the presence of a meniscal ramp lesion in stepwise forward multiple logistic regression analysis

Factor	Regression coefficient (*ß*)	SE	OR (95% CI)	*P* value
Contact sports	0.91	0.32	2.50 (1.32–4.72)	0.005
Pivot-shift-type bone bruising	0.25	0.13	1.29 (1.01–1.67)	0.04
PMT bone bruising	1.53	0.29	4.62 (2.61–8.19)	< 0.001
Segond fracture	1.48	0.58	4.38 (1.40–13.68)	0.001

*CI* confidence intervals, *PMT* posteromedial tibial, *OR* odds ratio, *SE* standard error

## Discussion

The most important finding in this study was that the prevalence of meniscal ramp lesions in patients with an ACL injury was high (39.5%). This study also revealed the prevalence of the different meniscal ramp lesion types. Finally, another important finding was that meniscal ramp lesions were associated with contact sports at ACL injury and the presence on MRI of pivot-shift-type bone bruising, PMT bone bruising and a Segond fracture.

Previous studies have reported a variable prevalence (9.3%–40%) of meniscal ramp lesions diagnosed arthroscopically at the time of ACLR [6, 11, 26, 36]. This wide variation in prevalence might depend on the different definitions of ramp lesions employed [2, 6, 11, 18, 26, 36], as well as, which is probably more important, the method employed for diagnosis. Although direct arthroscopic visualisation is regarded as the gold standard for diagnosing ramp lesions [6, 8, 26, 36], several studies have shown that the standard anteromedial and anterolateral portal have low sensitivity when diagnosing ramp lesions [19, 28, 36]. While inspection through the Gillquist view, the use of a 70-degree arthroscope (while viewing the posteromedial ramp area) and, more importantly, the creation of a posteromedial portal are more accurate in detecting ramp lesions [7, 19, 36, 39, 44], they are not routinely used. This may have led to an underestimation of the true incidence of meniscal ramp lesions in the literature. Sonnery-Cottet et al. [36] systematically explored the posterior horn of the MM in three sequential stages. In the first stage, exploration was performed through standard anterior visualisation via the anterolateral portal. In the second stage, the posterior horn of the MM was visualised through the Gillquist view. Finally, in the third stage, the posterior horn was probed through an additional posteromedial portal. The authors reported the highest (40%) prevalence of meniscal ramp lesions, diagnosed arthroscopically, in the literature. However, only 58% of meniscal ramp lesions were diagnosed at the second stage, with inspection through the Gillquist view. Forty-two per cent were only diagnosed at the third stage, after probing and debridement with a motorised shaver of a superficial tissue layer over the meniscocapsular junction covering the “hidden lesion”. Despite using MRI as a diagnostic method, the prevalence of meniscal ramp lesions in the present series is comparable to that of Sonnery-Cottet et al. [36] (39.5% vs. 40%, respectively). In their study, Balazs et al. [3] also utilised MRI to determine whether a meniscal ramp lesion was present. In line with our results, the overall prevalence of meniscal ramp lesions in their series was 42% [3]. As suggested by Sonnery-Cottet et al. [36], it might be argued that, without the creation of a posteromedial portal and superficial soft tissue dissection over the meniscocapsular junction, many meniscal ramp lesions may be missed. Studies investigating the prevalence of meniscal ramp lesions without the systematic creation of a posteromedial portal and tissue debridement at the meniscocapsular junction reported a prevalence between 15.5% and 24% [11, 25, 26, 34, 40, 41]. This corresponds roughly to the prevalence (23.2%) of meniscal ramp lesions identified by Sonnery-Cottet et al. [36] using only exploration through the Gillquist view. Previous literature may have missed a significant number of meniscal ramp lesions that Sonnery-Cottet et al. [36] identified after soft tissue debridement through the posteromedial portal and we, as well as Balazs et al. [3], identified with MRI. MRI has high (> 92%) specificity for diagnosing ramp lesions when compared with probing through the posteromedial portal [2].

The use of MRI for the diagnosis of meniscal ramp lesions has been criticised due to the moderate sensitivity reported in previous literature [2, 11, 16, 19, 28, 41]. However, studies evaluating the accuracy of MRI for diagnosing meniscal ramp lesions (with arthroscopy as a gold standard) used different pathological signs to define these tears [2, 11, 20, 25, 41, 43]. In several studies [2, 11, 20, 41] a meniscal ramp lesion on MRI was only defined by a tear in the peripheral attachment of the posterior horn of the medial meniscus at the meniscocapsular junction. Other studies did not even report which MRI criteria were used for the diagnosis of meniscal ramp lesions [6, 28, 40]. Failure to consider the different types of meniscal ramp lesion and their varied MRI appearance [14] may have been responsible for an underestimation of the real prevalence of these injuries as well as for the reported reduced sensitivity of this imaging modality.

Bollen et al. [6] found a meniscal ramp lesion in 17 of a series of 183 ACLRs. The MRI was performed on 11 patients with a meniscal ramp lesion, but it was unable to identify the injury in any case. However, the study did not report which MRI criteria were used for the diagnosis, who read the MRI scans and with which experience and which magnetic field strength and sequences were used. Moreover, the time from injury to MRI was not reported. The author attributed the poor sensitivity of MRI in diagnosing ramp lesions to the fact that this examination is performed with the knee in near full extension and, as a result, the meniscocapsular separation is probably reduced, leading to false negatives. This theory has subsequently been supported by other authors [11, 25, 41]. However, a short time from injury to MRI (19.6 ± 15.2 days, in the present study) may prevent oedema in the injured structures of the posteromedial ramp area to reabsorb, allowing the diagnosis of meniscal ramp lesions regardless of the position of the knee. MRI performed with appropriate magnetic field strength and spatial resolution allows the clear visualisation of the entire thickness of the meniscal ramp area [3].

Several studies have reported a higher prevalence of meniscal ramp lesions in the event of the ACL tear being caused by a contact injury [3, 27, 34, 35]. Even if information about the exact injury mechanism was not available in the present study, our findings are somewhat in line with previous literature, as meniscal ramp lesions were associated with contact sports at ACL injury. As suggested by Seil et al. [34], it might be hypothesised that meniscal ramp lesions are more common in the event of high-energy trauma. This might also support the association between meniscal ramp lesions and pivot-shift bone bruising found in this study. Bisson et al. [5] showed that contact injuries were associated with more severe bone bruising in the lateral tibial plateau and that the increased severity of lateral tibial plateau bone bruising was associated with medial meniscal tears.

In the present study, PMT bone bruising was the factor with the strongest association with meniscal ramp lesions (OR 4.62; 95% CI 2.61–8.19; *P* < 0.001). This MRI sign was present in 61% of the patients with meniscal ramp lesions in comparison with 25.5% without. These findings contrast with those of Song et al. [35] and Hatayama et al. [16] reporting that PMT bone bruising is not associated with meniscal ramp lesions. These differences might be related to the different timing of MRI. Hatayama et al. [16] reported a time interval between injury and MRI ranging from 1 day to 10 years, whereas Song et al. [35] did not report the delay from injury to MRI. In the present study, the short delay (19.6 ± 15.2 days) from injury to MRI may have prevented the PMT bone bruising to reabsorb and therefore increased its association with meniscal ramp lesions. Most of the literature suggests that PMT bone bruising is an important secondary MRI sign of meniscal ramp lesions [3, 4, 11, 20, 22, 23, 41]. The strong association between PMT bone bruising and meniscal ramp lesions might be due to one possible common injury mechanism. A contrecoup injury with impaction of the MFC and PMT plateau, due to a sudden tibial reduction with compensatory varus alignment and internal tibial rotation after the initial pivot-shift mechanism, might be the origin of both meniscal ramp lesions and PMT bone bruising [11, 17]. The same consideration might be applied to the Segond fracture, which is thought to occur as the result of internal rotation and varus stress [9, 33]. This injury was strongly associated (OR 4.38; 95% CI 1.40–13.68; *P* = 0.001) with the presence of meniscal ramp lesions.

Meniscal ramp lesions are more common than previously thought. The findings in the present study suggest that they are among the most frequent injuries associated with ACL tears. Surgeons who treat ACL tears are likely to encounter meniscal ramp lesions in daily practice. The recognition of meniscal ramp lesions is essential, as they are associated with increased anterior and rotational laxity, increased strain on both the native and ACL graft, as well as accelerated cartilage degeneration in the medial compartment [6, 12, 15, 29, 30, 37]. Only the repair of meniscal ramp lesions is able to restore anterior and rotational laxity [12, 37]. If meniscal ramp lesions are overlooked in patients with ACLR, anterior and rotational laxity persists [1, 7, 12, 37].

This study provides important information regarding the prevalence and appearance of MRI of meniscal ramp lesions in patients with ACL injuries. In addition, it identifies some factors (contact sport at ACL injury, pivot-shift-type bone bruising, PMT bone bruising and a Segond fracture) associated with meniscal ramp lesions. Their presence should further raise surgeons’ and radiologists’ suspicion of these important tears.

The main strength of this study was that the evaluation of meniscal ramp lesions was performed using standardised and well-defined MRI pathological signs [14]. An orthopaedic surgeon specialising in knee surgery and a musculoskeletal radiologist reviewed all the MRI scans. Moreover, a substantial number (54.5%) of MRI examinations were performed using a 3.0 Tesla scanner. These factors probably improved the diagnosis of meniscal ramp lesions [21]. The short delay (19.6 ± 15.2 days) from the injury to the MRI may have prevented oedema in the injured structures of the posteromedial ramp area to reabsorb and therefore increased the accuracy of MRI in diagnosing meniscal ramp lesions. In addition, it strengthened the association between bone bruises (that would otherwise have reabsorbed over time) and meniscal ramp lesions. Finally, the cohort studied and the number of patients with meniscal ramp lesions were relatively large (*n* = 253 and 100, respectively). This enabled the analysis of several factors potentially associated with the presence of meniscal ramp lesions in our logistic regression analysis.

There are some limitations. MRI examinations were performed at different institutions with different scanners. However, the imaging protocol was standardised and all the MRI scans were performed at 1.5 or 3.0 Tesla and were reviewed by the same orthopaedic surgeon and musculoskeletal radiologist. Other factors, such as medial posterior tibial slope, medial meniscal slope, gradual lateral tibial slope and varus alignment of more than three degrees, were not controlled for, although they were previously associated with the presence of meniscal ramp lesions [20, 35]. However, also analysing these factors would have required a much larger number of patients.

## Conclusion

The overall prevalence of meniscal ramp lesions in patients with ACL injuries was high (39.5%). Contact sports at ACL injury, pivot-shift-type bone bruising, PMT bone bruising and the presence of a Segond fracture on MRI were associated with meniscal ramp lesions. Given their high prevalence, meniscal ramp lesions should be systematically searched for on MRI in patients with ACL injuries. Knowledge of the factors associated with meniscal ramp lesions may facilitate their diagnosis, raising surgeons’ and radiologists’ suspicion of these tears.
